# Brain Dynamics Altered by Photic Stimulation in Patients with Alzheimer’s Disease and Mild Cognitive Impairment

**DOI:** 10.3390/e23040427

**Published:** 2021-04-04

**Authors:** Wei-Yang Yu, Intan Low, Chien Chen, Jong-Ling Fuh, Li-Fen Chen

**Affiliations:** 1 Institute of Brain Science, National Yang Ming Chiao Tung University, Taipei 112, Taiwan; justerxjapg@gmail.com (W.-Y.Y.); itlow@nycu.edu.tw (I.L.); 2 Integrated Brain Research Unit, Department of Medical Research, Taipei Veterans General Hospital, Taipei 112, Taiwan; 3 Department of Neurology, Neurological Institute, Taipei Veterans General Hospital, Taipei 112, Taiwan; cchien0604@gmail.com; 4 Faculty of Medicine, College of Medicine, National Yang Ming Chiao Tung University, Taipei 112, Taiwan; 5 Brain Research Center, National Yang Ming Chiao Tung University, Taipei 112, Taiwan; 6 Institute of Biomedical Informatics, National Yang Ming Chiao Tung University, Taipei 112, Taiwan

**Keywords:** Alzheimer’s disease, mild cognitive impairment, photic stimulation, multiscale sample entropy, electroencephalography, EEG complexity, brain dynamics, brain adaptability

## Abstract

Individuals with mild cognitive impairment (MCI) are at high risk of developing Alzheimer’s disease (AD). Repetitive photic stimulation (PS) is commonly used in routine electroencephalogram (EEG) examinations for rapid assessment of perceptual functioning. This study aimed to evaluate neural oscillatory responses and nonlinear brain dynamics under the effects of PS in patients with mild AD, moderate AD, severe AD, and MCI, as well as healthy elderly controls (HC). EEG power ratios during PS were estimated as an index of oscillatory responses. Multiscale sample entropy (MSE) was estimated as an index of brain dynamics before, during, and after PS. During PS, EEG harmonic responses were lower and MSE values were higher in the AD subgroups than in HC and MCI groups. PS-induced changes in EEG complexity were less pronounced in the AD subgroups than in HC and MCI groups. Brain dynamics revealed a “transitional change” between MCI and Mild AD. Our findings suggest a deficiency in brain adaptability in AD patients, which hinders their ability to adapt to repetitive perceptual stimulation. This study highlights the importance of combining spectral and nonlinear dynamical analysis when seeking to unravel perceptual functioning and brain adaptability in the various stages of neurodegenerative diseases.

## 1. Introduction

Alzheimer’s disease (AD) is an irreversible neurodegenerative disorder and the most prevalent form of dementia [1]. Brain deterioration in AD patients is characterized by the accumulation of β-amyloid plaques and neurofibrillary tangles consisting of tau amyloid fibrils, which can cause local neuronal death, neurotransmitter deficiencies [2], and cortical disconnection [3]. As a result, AD patients suffer from declining memory and cognitive functions as well as functional disabilities. Mild cognitive impairment (MCI) is a clinical neuropsychological syndrome characterized by cognitive and memory impairments [4]. Most MCI patients retain the functional abilities required to perform daily activities; however, they face an elevated risk for AD, with a progression rate of 10% to 15% per year [5]. Magnetic resonance imaging (MRI) and positron emission tomography (PET) are commonly used to investigate the neuropathology in AD and MCI patients [6,7]; however, these methods are costly and inaccessible to many patients. In the current study, we sought to develop the means to rapidly differentiate between AD and MCI using simple examinations that do not require highly specialized equipment or training.

Electroencephalography (EEG) is a non-invasive and relatively low-cost neuroimaging technique that records scalp electrical signals generated by the synchronous activity of pyramidal neurons in the brain. EEG provides outstanding temporal resolution by which to capture brain dynamics and neural oscillations in real-time. The brain is a highly complex system [8], and neural activity is nonlinear and non-stationary [9]. Rhythmic EEG activity can be characterized using linear methods (e.g., power spectral analysis); however, nonlinear methods have proven particularly effective in exploring brain dynamics and complexity. Complexity represents “meaningful structural richness” [10], and entropy is among the most common nonlinear approach to analyzing signal complexity [11]. Sample entropy (SE) [12], an information theory-based metric, was proposed to overcome the weaknesses of its precedence, the approximate entropy [13], including the bias of self-matches, relative inconsistency, and dependence on large data points and sample length. SE computes the degree of similarity between two sequences in order to characterize the uncertainty and unpredictability in physiological time-series signals [14]. As such, signals with repetitive structures (such as rhythmic oscillations) are more regular, predictable, and would yield low SE values; signals with random structures (such as random noise) are more irregular, unpredictable, and would yield high SE values. However, high SE values quantified from noise do not represent meaningful complexity; the SE value at a single time scale could not fully capture the hierarchical and dynamical spatial and temporal structures in a real, complex physiological system [15]. As an extension, multiscale sample entropy (MSE) [10] calculates SE values within each coarse-grained time series in order to account for signal complexity over multiple temporal scales, from small (or “short” or “fine”) scales that reflect microscopic and local dynamics to large (or “long” or “coarse”) scales that reflect macroscopic and long-range dynamics [16,17,18], thereby making it possible to differentiate meaningful complexity from random noise [10].

Resting-state brain signals reflect spontaneous brain activities manifesting intrinsic mental states and changes brought about by diseases. Under resting-state conditions, many AD patients present increased low-frequency EEG power and decreased high-frequency EEG power. It has been posited that this is the result of alpha slowing due to thalamocortical dysrhythmia [19,20,21,22,23,24]. In order to investigate changes in nonlinear intrinsic brain complexity, MSE-based analyses have been applied to resting-state EEG and MEG signals of individuals with different pathological conditions [9,25], such as psychiatric disorders [26,27], pain conditions [28,29,30,31], and neurological diseases [32,33,34,35,36,37,38]. In particular, MSE-based resting-state EEG studies in AD and dementia patients [33,34,35,36,37,39,40,41] have reported that at small time scales, entropy values were lower in MCI and AD patients than in healthy controls; at large time scales, entropy values tended to be higher in AD patients than in healthy controls. Mizuno et al. [35] concurrently investigated EEG complexity and relative EEG power ratio using eyes-closed resting-state EEG signals in AD patients and healthy elderly controls. AD patients showed reduced complexity at smaller scales in frontal regions and higher complexity at larger scales across the brain that were related to cognitive decline. In addition, MSE values at smaller and larger scales were respectively associated with higher and lower frequencies of relative EEG power. Other studies found that the slope of MSE at large scales was smaller in AD patients than in healthy controls [42,43] and might potentially predict the efficacy of acetylcholinesterase inhibitors for AD treatment [42]. These findings suggest that abnormal resting-state brain dynamics are related to AD severity and might therefore be identifiable via MSE analysis [34,35,36,39]. Note, however, that resting-state brain dynamics alone is insufficient to characterize the adaptive changes in the brain in response to a changing environment. Thus, in seeking to understand the functional adaptability in AD and MCI patients, it is also important to study the means by which brain systems cope with perturbations due to external stimulation.

Intermittent rhythmic photic stimulation (PS) is widely employed in clinical EEG examinations targeting perceptual functions, such as visual functions [44,45]. In a healthy system, the brain gradually entrains to the frequency of repetitive visual stimuli, leading to an increase in EEG rhythmic power [46] and a decrease in the complexity of EEG signals [47]. Takahashi et al. [48] reported that after undergoing PS, young adults presented significant increases in EEG complexity, whereas elderly subjects presented no changes in complexity, indicating flexible brain adaptability in a healthy resilient brain and a loss of complexity in an aging brain. Patients with neurodegenerative diseases suffer from a more severe form of neuronal degeneration than their healthy counterparts. In previous studies, induced EEG oscillatory power in response to PS (5-Hz, 10-Hz, and 15-Hz) was lower in patients with AD than in healthy controls [49,50]. These findings suggest that perceptual functioning in AD patients are prone to disruption.

To the best of our knowledge, this is the first study to investigate perceptual functioning and brain adaptability before, during, and after intermittent photic stimulation in patients with mild AD, moderate AD, severe AD, and MCI as well as healthy elderly subjects. We evaluated PS-induced alterations in neural oscillatory responses and brain dynamics using linear analysis (power spectrum density) as well as nonlinear analysis (MSE). We hypothesized that perceptual functioning and brain adaptability deteriorate with disease progression. Thus, we expected that during PS, neural oscillatory responses would be lower in AD patients than in healthy controls and brain dynamics would appear more irregular. We also hypothesized that within-group PS-induced changes (before, during, and after PS) in EEG complexity would vary in accordance with disease severity.

## 2. Materials and Methods

### 2.1. Subjects

In this study, we included a subset of the participants from the multimodal biosignature study in AD in Taiwan. The protocol was approved by the Institutional Review Board of Taipei Veterans General Hospital (IRB number: 2012-05-033B) and conducted in accordance with the Declaration of Helsinki. Written informed consent forms were signed by all the participants before the study. Participants were individuals recruited from outpatient settings, between 45 to 90 years old, and had normal vision and hearing abilities. Participants underwent neuropsychological examinations and assessments, including Mini-Mental State Examination (MMSE) and Clinical Dementia Rating (CDR) scale, laboratory tests, neuroimaging evaluation, and EEG monitoring. Clinical diagnosis was performed by neurologists, and the inclusion criteria were as follows. An AD diagnosis was made according to the clinical criteria for probable AD as described by the National Institute on Aging–Alzheimer’s Association (NIA-AA) [51,52]. A diagnosis of MCI was made according to the revised consensus criteria from 2004 [53,54] and Petersen’s criteria for MCI [54,55,56]. When the cognitive symptoms of patients significantly interfered with their abilities or work functions, which were evaluated through a combination of history-taking and cognitive assessment, they were diagnosed with dementia. The cut-off value for the diagnosis of MCI was set at 1.5 standard deviations below the age-adjusted norm for the logical memory test of the Wechsler Memory Scale-III. Participants received their regular medications or routine treatments. The exclusion criteria were subjects who had significant neurological diseases other than AD that may affect cognition, including Parkinson’s disease, vascular dementia, normal pressure hydrocephalus, brain tumor, progressive supranuclear palsy, seizure disorder, subdural hematoma, multiple sclerosis, or a history of significant head trauma followed by permanent neurological deficits or known structural brain abnormalities.

Data from 134 subjects were included in this EEG study: 80 AD patients (43 males, 37 females; age = 75.85 ± 9.16 years old), 34 MCI patients (15 males, 19 females; age = 68.4 ± 6.4 years old), and 20 healthy elderly controls who presented normal cognitive functions (HC; 10 males, 10 females; age = 71.0 ± 5.5 years old). AD severity was rated in terms of MMSE scores. AD patients (MMSE ≤ 24) were categorized into three subgroups: Mild AD (MMSE = 20–24; *N* = 38), Moderate AD (MMSE = 14–19; *N* = 31), and Severe AD (MMSE ≤ 13; *N* = 11). Throughout this article, “non-AD groups” refers to the HC and MCI groups, whereas “AD subgroups” refers to the Mild, Moderate, and Severe AD groups.

### 2.2. EEG Recording and Data Pre-Processing

All subjects underwent a routine clinical EEG recording (NicoletOne EEG, Natus Medical Incorporated, San Carlos, CA, USA) in the EEG examination room at the Institute of Neurology, Taipei Veterans General Hospital. Before EEG recording, skin preparation was done by gently scrubbing the subject’s scalp with cotton tips and cleansing gel. A small amount of paste (Ten20 EEG Conductive Paste, Weaver and company, Colorado, USA) was put into the cup of each electrode, and a small square of gauze was covered over each electrode. Twenty-five single EEG electrodes (Genuine Grass^®^ reusable EEG cup electrodes, Natus Medical Incorporated) were manually attached to the scalp in accordance with the international 10–20 system, including Fp1, F3, F7, C1, C3, T3, T5, A1, P3, O1, Fp2, F4, F8, C2, C4, T4, T6, A2, P3, O2, Fpz, Fz, Fcz, Cz, and Pz. The reference electrode was Fcz, and the ground electrode was Fpz. EEG impedance threshold was below 5 kOhms, and EEG recordings were obtained at a sampling rate of 1000 Hz. Heart rate was also recorded for artifact rejection.

**Figure 1:**
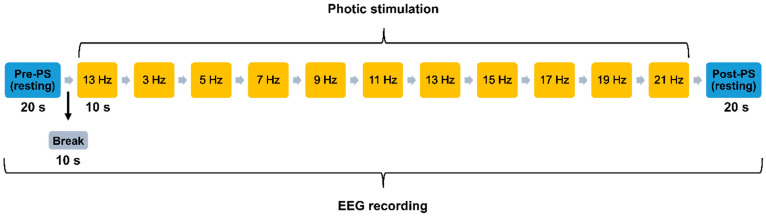
Photic stimulation (PS) session performed during routine clinical EEG examination. Pre- and Post-PS resting-state EEG signals were recorded. PS involved flashing a white light for 10 s at various frequencies with 10-s breaks in between. Subjects kept their eyes closed throughout the EEG recording.

Throughout the EEG examination, the subjects sat on a chair with their eyes closed in a dimly lit room. This routine clinical EEG examination consisted of resting-state, hyperventilation, intermittent photic stimulation (IPS) [44], and sleep conditions. Resting-state conditions included one 20-s eyes-closed resting-state EEG recording before PS (“Pre-PS”) and one after PS (“Post-PS”). In this study, we focused on the PS condition, in which subjects were exposed to white light flashing at frequencies of 3, 5, 7, 9, 11, 13, 15, 17, 19, and 21 Hz (see Figure 1) with their eyes closed. Each PS frequency was administered for a period of 10 s (“during PS”) and was preceded by an interval of 10 s (“break”).

EEG signal pre-processing and analysis were performed in the Matlab environment (R2018b version, MathWorks, Natick, MA, USA) using the Brainstorm toolbox [57]. Data pre-processing involved re-referencing all EEG channels using the average of the right and left mastoids. Raw EEG data were resampled to 250 Hz and filtered to 2–50 Hz. Heartbeat artifacts were removed using the signal space projection (SSP) method. This study targeted PS-induced alterations in brain complexity and perceptual functioning; therefore, the channels of interest included the O1, O2, P3, and P4 electrodes targeting the visual cortex.

### 2.3. EEG Power Analysis

Each 10-s EEG signal during PS was segmented into five non-overlapping 2-s epochs. Absolute EEG power at the three fundamental PS frequencies representing theta, alpha, and beta bands (5 Hz, 9 Hz, and 15 Hz) and the harmonics of fundamental frequencies up to 45 Hz were estimated using time-frequency Morlet wavelet analysis. Estimates of EEG power values were obtained for the following frequencies: 5-Hz PS (5, 10, 15, 20, 25, 30, 35, and 45 Hz), 9-Hz PS (9, 18, 27, 36, and 45 Hz), and 15-Hz PS (15, 30, and 45 Hz). The absolute EEG power values of the five epochs were then averaged to yield an average absolute EEG power for each frequency. Using the same procedure, absolute EEG power estimates were also obtained for the 10-s break preceding each PS. 

EEG power ratios were used as indices of oscillatory responses. These were calculated as the average absolute EEG power values at *f*∗*n*-Hz during a given *f*-Hz PS divided by EEG power values obtained during the 10-s break preceding that *f*-Hz PS (“PS/break”), as follows:

(1) $$\mathrm{EEG}\;\mathrm{power}\;\mathrm{ratio}\;\mathrm{at}\;f*n\;\mathrm{Hz}\;=\;\frac{\mathrm{Average}\;\mathrm{absolute}\;\mathrm{EEG}\;\mathrm{power}\;\mathrm{at}\;f*n\;\mathrm{Hz}\;\mathrm{during}\;f-\mathrm{Hz}\;\mathrm{PS}}{\mathrm{Average}\;\mathrm{absolute}\;\mathrm{EEG}\;\mathrm{power}\;\mathrm{at}\;f*n\;\mathrm{Hz}\;\mathrm{during}\;\mathrm{the}\;10-s\;\mathrm{break}\;\mathrm{before}\;f-\mathrm{Hz}\;\mathrm{PS}}$$

where *f* = 5, 9, 15 Hz (i.e., three fundamental frequencies of PS) and *n* = 1, 2, …, *f* ∗*n* < 45 (*n* = number of harmonics).

### 2.4. EEG Multiscale Sample Entropy (MSE) Analysis

MSE analysis is used to quantify the complexity of physiologic signals in multiple temporal series. Two parameters, *m* and *r,* are required to calculate sample entropy (SE) values [12], where *m* refers to the length of the patterns to be compared, and *r* refers to the amplitude threshold used to designate similarity between two vectors. Given an EEG signal containing *N* sample points, $x=\left[x_{1},x_{2},\cdots ,x_{N}\right]$.

Two vectors of length *m* are defined as follows:

(2) $$X_{m}\left(i\right)=\left[x_{i},x_{i+1},\;\cdots ,\;x_{i+m-1}\right],\;\mathrm{where}\;i=1,\cdots ,N-m$$

(3) $$X_{m}\left(j\right)=\left[x_{j},x_{j+1},\;\cdots ,\;x_{j+m-1}\right],\;\mathrm{where}\;j=1\leq j\leq N-m\;\mathrm{and}\;j\neq m$$

The distance between vectors $X_{m}\left(i\right)$ and $X_{m}\left(j\right)$ is calculated as follows:

(4) $$dist\left(X_{m}\left(i\right),X_{m}\left(j\right)\right)=\|x_{i+k-1}-x_{j+k-1}\|$$

SE is defined as follows:

(5) $$SampEn=-\ln\frac{A}{B}$$

where *A* indicates the number of template vector pairs in which $dist\left(X_{m+1}\left(i\right),\;X_{m+1}\left(j\right)\right)<r$, and *B* indicates the number of template vector pairs in which $dist\left(X_{m}\left(i\right),X_{m}\left(j\right)\right)<r$. 

Calculating SE of different time scales involves coarse-graining the original EEG time series $\left\{x_{1},\;x_{2,\;}\cdots ,x_{N}\right\}$ using different time-scale factors [10] in non-overlapping windows, as follows:

(6) $$y_{j}^{\left(\tau \right)}=\left(\frac{1}{\tau }\right)\sum_{i=\left(j-1\right)\tau +1}^{j\tau }x_{i},\;1\leq j\leq N/\tau ,$$

where *τ* is the scale factor (SF) of the time series. SE calculation is then applied to each time series to produce MSE values of all scale factors.

This analysis was performed using the multiscale sample entropy toolbox developed by the Laboratory of Precision Psychiatry (http://www.psynetresearch.org/, accessed on 1 April 2021). The number of sampling points during each 10-s PS were *N* = 2500 (10 s × 250 Hz), and the number of sampling points during each 20-s Pre-PS and Post-PS resting states were *N* = 5000 (20 s × 250 Hz). In accordance with previous EEG studies using MSE analysis for AD [35,48], the parameters were set as follows: *m* = 2, *r* = 0.2, and *τ* = 1 to 20. Note that in this study, the term “small scales” refers to SF 1 to 7 and “large scales” refers to SF 8 to 20.

### 2.5. Statistical Analysis

SPSS (Windows version 22; IBM Inc., Armonk, NY, USA) software was used for all statistical analyses, and a two-tailed α level of 0.05 was considered statistically significant. This study included the following five groups: Healthy elderly control (HC), MCI, Mild AD, Moderate AD, and Severe AD groups. For demographic data, one-way analysis of variance (ANOVA) was used to test group effects on age, education, and MMSE. Chi-square test was used to test the independent binomial proportions of gender in each group. The Bonferroni test was used for post-hoc multiple comparisons.

Channel-wise analysis was performed on EEG power ratios and MSE values. Scale-wise analysis was further performed on MSE values because SE differences between different scale factors were not of our interest. First, group differences among five groups were tested respectively for EEG power ratios during PS, resting-state MSE before PS (Pre-PS), MSE under the effects of PS (During PS), and resting-state MSE after PS (Post-PS) using one-way ANCOVAs with age as the covariate, and the Bonferroni test was used for post-hoc multiple comparisons. Second, we were particularly interested in the changes in brain dynamics altered by PS in each group. Therefore, within-group MSE differences among Pre-PS, during PS, and Post-PS were tested in each group using one-way repeated measures ANOVAs (Freidman test was used for the Severe AD group; *N* = 11), and the Bonferroni test was used for post-hoc multiple comparisons. Third, to explore the relationship between nonlinear brain dynamics and linear oscillatory responses during PS, Spearman’s rank correlations were used to calculate the correlation coefficients between MSE values and EEG power ratio values during PS. To avoid exhaustive computation in exploring correlations, only EEG power ratios and MSE values that showed significant group effects during PS (one MSE value averaged for SF 9 to 14 and one for SF 15 to 20) were included in the correlation analysis.

## 3. Results

### 3.1. Demographics and MMSE Scores

Table 1 lists the demographic data and MMSE results. We observed no significant gender effects. We observed significant group effects on age (*F*(4, 129) = 6.586, *p* < 0.001) and education level (*F*(4, 129) = 3.696, *p* < 0.01). Post-hoc comparisons revealed that the HC group was significantly younger than the Moderate AD group (Bonferroni-corrected *p* = 0.035), and the MCI group was significantly younger than the Mild AD (Bonferroni-corrected *p* = 0.005) and Moderate AD (Bonferroni-corrected *p* < 0.001) groups. Therefore, age was considered a confounding factor and a covariate in ANCOVA in assessing EEG data. The education level in the Moderate AD group was significantly lower than in the MCI group (Bonferroni-corrected *p* = 0.021). 

We observed a significant group effect on MMSE scores (*F*(4, 129) = 297.070, *p* < 0.001). Post-hoc comparisons revealed no significant differences between the two non-AD groups (HC and MCI) (Bonferroni-corrected *p* = 0.117). However, the MMSE scores of both non-AD groups were significantly higher (better) than those in the AD subgroups (Bonferroni-corrected *p* < 0.0001). MMSE scores decreased significantly in the following order: Severe AD < Moderate AD < Mild AD (Bonferroni-corrected *p* < 0.0001).

### 3.2. EEG Power Ratios of Harmonic Responses during PS Were Lower in AD Subgroups

**Figure 2:**
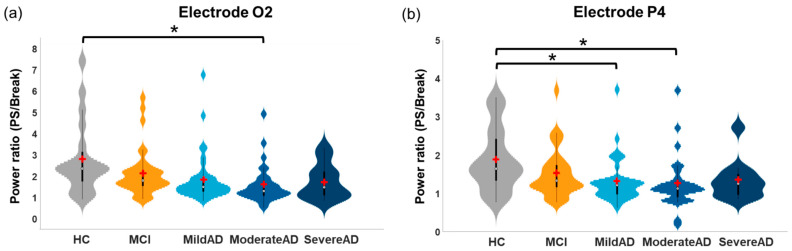
Significant between-group differences in EEG power ratios of 36-Hz harmonic responses to 9-Hz PS at (**a**) electrode O2 and (**b**) electrode P4 (* Bonferroni-corrected *p* < 0.05). Distribution plots integrated with box plots are presented; violin plots represent the distributions of the data, black boxes represent interquartile ranges (IQR; 25th to 75th percentiles), white circles in each box indicate medians (50%), and red crosses indicate means.

No significant group effects were observed for EEG power ratios associated with the fundamental PS frequencies (5-Hz, 9-Hz, and 15-Hz). Significant group effects were observed for 20-Hz harmonic responses to 5-Hz PS at the O1 electrode (*F*(4, 125) = 3.453, *p* = 0.01), and for 25-Hz harmonic responses (*F*(4, 125) = 3.763, *p* = 0.006) and 30-Hz harmonic responses (*F*(4, 127) = 2.981, *p* = 0.022) to 5-Hz PS at the O2 electrode. Significant group effects were observed for 36-Hz harmonic responses to 9-Hz PS (Figure 2) at electrodes O2 (*F*(4, 128) = 2.547, *p* = 0.043) and P4 (*F*(4, 128) = 2.626, *p* = 0.038). Post-hoc comparisons revealed that compared to the HC group, EEG power ratios were significantly lower in the Moderate AD group (Bonferroni-corrected *p* = 0.0038) at the O2 electrode (Figure 2a) as well as in the Mild AD group (Bonferroni-corrected *p* = 0.043) and Moderate AD group (Bonferroni-corrected *p* = 0.048) at the P4 electrode (Figure 2b).

**Figure 3:**
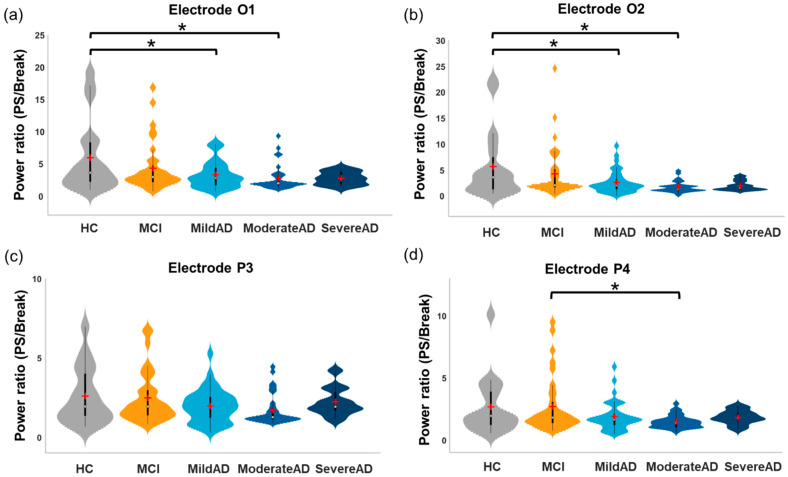
Significant between-group differences in EEG power ratios of 30-Hz harmonic responses to 15-Hz PS at electrodes (**a**) O1, (**b**) O2, (**c**) P3, and (**d**) P4 (* Bonferroni-corrected *p* < 0.05). Distribution plots integrated with box plots are presented; violin plots represent the distributions of the data, boxes represent interquartile ranges (IQR; 25th to 75th percentiles), white circles in each box indicate medians (50%), and red crosses indicate means.

Significant group effects were observed in the EEG power ratio of 30-Hz harmonic responses to 15-Hz PS (Figure 3) at electrodes O1 (*F*(4, 127) = 3.442, *p* = 0.01), O2 (*F*(4, 127) = 4.652, *p* = 0.002), and P4 (*F*(4, 126) = 2.754, *p* = 0.031). Post-hoc comparisons revealed no significant differences in the EEG power ratios between HC and MCI groups at any electrode. Compared to the HC group, EEG power ratios were significantly lower than in the Mild AD (Bonferroni-corrected *p* = 0.03–0.042) and Moderate AD (Bonferroni-corrected *p* = 0.003–0.012) groups at electrodes O1 (Figure 3a) and O2 (Figure 3b). EEG power ratios were also significantly lower in the Moderate AD group than in the MCI group (Bonferroni-corrected *p* = 0.0499) at the O2 electrode. Compared to the MCI group, EEG power ratios were significantly lower in the Moderate AD group (Bonferroni-corrected *p* = 0.049) at the P4 electrode (Figure 3d). Overall, AD subgroups exhibited lower harmonic responses to PS, which might reflect a general decrease in neural oscillatory response.

### 3.3. Higher MSE Values at Large Scales before and during PS in AD Subgroups

**Figure 4:**
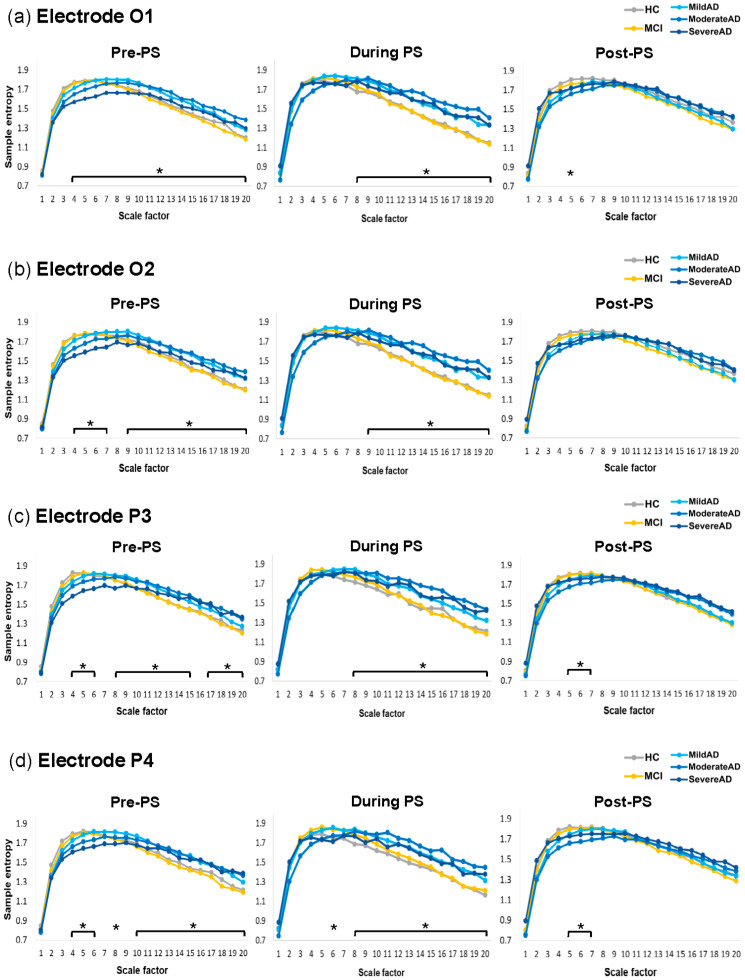
MSE profiles and between-group differences before PS (Pre-PS), under the effects of 15-Hz PS (During PS), and after PS (Post-PS) at electrodes (**a**) O1, (**b**) O2, (**c**) P3, and (**d**) P4. In each subfigure, lines in different colors are the mean SE values at each scale factor for each group (* *p* < 0.05, one-way ANCOVAs).

One-way ANCOVAs were run respectively to determine the effects of group on resting-state MSE Pre-PS, MSE during PS, and resting-state MSE Post-PS after controlling for age (Figure 4). Significant group differences were observed in MSE values from small to large scale factors before PS and at large scale factors during PS in all channels of interest, but group differences were mostly absent after PS. The AD subgroups did not present the distinct time scale-dependent patterns observed in the MSE profiles of the non-AD groups, in which the high initial MSE values at small scale factors dropped substantially at large scale factors.

Similar group differences were observed in the resting-state MSE values in all channels of interest both before PS and after PS; thus, the statistics of the representative O1 electrode were reported in detail. Before PS (Figure 4, left column), significant group effects were observed in resting-state MSE values at small (SF 4 to 7; *F*(4, 128) = 2.853–3.865, *p* = 0.005–0.026) and large scale factors (SF 8 to 20; *F*(4, 128) = 3.523–5.01, *p* = 0.009–0.001). Post-hoc results revealed that at small scale factors, MSE values were significantly lower in the Severe AD group than in the HC, MCI, and Mild AD groups (Bonferroni-corrected *p* = 0.004–0.037). At large scale factors, MSE values were significantly higher in the Mild AD and Moderate AD groups as follows: Mild AD > MCI (Bonferroni-corrected *p* = 0.002–0.049), Moderate AD > MCI (Bonferroni-corrected *p* = 0.001–0.016), and Moderate AD > HC (Bonferroni-corrected *p* = 0.018–0.035). 

The results of MSE group differences under the effects of 5-Hz PS (see Supplementary Materials, Figure S1), 9-Hz PS (see Supplementary Materials, Figure S2), and 15-Hz PS (Figure 4, middle column) are illustrated. Under the effects of 5-Hz PS (Figure S1), significant group effects were observed in MSE values at small (SF 4 and 5; *F*(4, 128) = 3.526–3.962, *p* = 0.005–0.009) and large scale factors (SF 10 to 20; *F*(4, 128) = 2.518–5.957, *p* = 0.0002–0.044) at electrodes O1 and O2. Significant group effects were also observed in MSE values at small (SF 3 to 8; *F*(4, 128) = 2.474–5.355, *p* = 0.0005–0.048) and large scale factors (SF 16 to 20; *F*(4, 128) = 2.754–4.937, *p* = 0.001–0.031) at electrodes P3 and P4. Overall, post-hoc results revealed that at small scale factors, MSE values were significantly lower in the AD subgroups than those in the HC and MCI groups (Bonferroni-corrected *p* = 0.001–0.049). At large scale factors, MSE values were significantly higher in the AD subgroups than in the HC and MCI groups (Bonferroni-corrected *p* = 0.001–0.049).

Under the effects of 9-Hz PS (Figure S2), significant group effects were observed in MSE values at large scale factors (SF 9 to 20) at electrodes O1 (*F*(4, 128) = 4.157–8.135, *p* = 0.000007–0.003), O2 (*F*(4, 128) = 3.619–6.972, *p* = 0.00004–0.008), P3 (*F*(4, 128) = 2.948–5.938, *p* = 0.0002–0.023), and P4 (*F*(4, 128) = 3.051–6.357, *p* = 0.0001–0.019). Post-hoc results revealed that MSE values were significantly higher in the AD subgroups than in the HC and MCI groups (Bonferroni-corrected *p* = 0.00012–0.047).

Under the effects of 15-Hz PS (Figure 4, middle column), the findings were similar to those obtained under 9-Hz PS. At large scale factors (SF 8 to 20), significant group effects were observed in MSE values at electrodes O1 (Figure 4a, middle column; *F*(4, 128) = 2.684 –6.593, *p* = 0.00007–0.034), O2 (Figure 4b, middle column; *F*(4, 128) = 4.377–7.960, *p* = 0.0000092–0.002), P3 (Figure 4c, middle column; *F*(4, 128) = 2.599–5.407, *p* = 0.0005–0.039), and P4 (Figure 4d, middle column; *F*(4, 128) = 2.443–5.504, *p* = 0.0004–0.05). Post-hoc results of electrode O1 revealed that MSE values in the Moderate AD group were significantly higher than those in the HC group (Bonferroni-corrected *p* = 0.001–0.033) and MCI group (Bonferroni-corrected *p* = 0.0002–0.013). Furthermore, MSE values in the Mild AD group were significantly higher in the HC group (Bonferroni-corrected *p* = 0.022) and MCI group (Bonferroni-corrected *p* = 0.026–0.036).

### 3.4. Distinct Patterns in PS-Induced Changes in MSE Values in AD and Non-AD Groups

**Figure 5:**
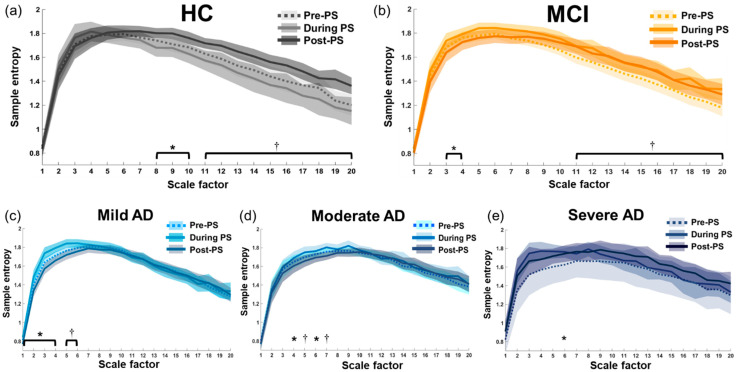
MSE profiles and PS-induced changes (Pre-PS, during 15-Hz PS, and Post-PS) in each group at the representative O1 electrode: (**a**) Healthy control, (**b**) Mild cognitive impairment group, (**c**) Mild AD group, (**d**) Moderate AD group, and (**e**) Severe AD group. In each subfigure, each line plots the mean SE values at each scale factor under each condition (Pre-PS, During PS, Post-PS). The shaded error area surrounding each line shows its 95% confidence interval. (* Bonferroni-corrected *p* < 0.05, † Bonferroni-corrected *p* < 0.01).

Similar patterns were observed in the within-group PS-induced changes (Pre-PS, During PS, and Post-PS) in MSE values in all channels of interest. Overall, PS-induced changes were ranked as follows: HC > MCI > Mild AD > Moderate AD > Severe AD. Figure 5 presents the MSE profiles and PS-induced changes in each group at the representative O1 electrode; the statistics of electrode O1 were reported in detail.

PS-induced changes in brain dynamics were most prominent in the non-AD groups at large scale factors. The HC group (Figure 5a) presented significantly lower MSE values during PS (Bonferroni-corrected *p* = 0.001–0.026) and before PS (Bonferroni-corrected *p* = 0.001–0.046) compared to after PS at large scale factors (SF 8 to 20). Similar to the HC group, the MCI group (Figure 5b) also presented significantly lower MSE values during PS (Bonferroni-corrected *p* = 0.00004–0.003) and before PS (Bonferroni-corrected *p* = 0.035–0.043) compared to after PS at large scale factors (SF 11 to 20). However, unlike the HC group, the MCI group presented significantly higher MSE values during PS than after PS (Bonferroni-corrected *p* = 0.0018–0.003) at small scale factors (SF 3 to 4), which was similar to the AD subgroups.

In all three AD subgroups, PS-induced changes were absent at large scale factors. Most of the changes were at small scale factors, which were significantly higher during PS, ranked as follows: During PS > Pre-PS > Post-PS. The Mild AD group (Figure 5c) presented significantly higher MSE values during PS compared to before PS (Bonferroni-corrected *p* = 0.001–0.007) and after PS (Bonferroni-corrected *p* < 0.000001–0.004) at small scale factors (SF 1 to 6), and significantly higher MSE values before PS than after PS (Bonferroni-corrected *p* = 0.01–0.042) at small scale factors (SF 1 to 4). The Moderate AD group (Figure 5d) presented higher MSE values during PS than after PS (Bonferroni-corrected *p* = 0.001–0.017) at small scale factors (SF 4 to 7). In the Severe AD group (Figure 5e), PS-induced changes were almost absent, with only one significantly higher MSE value during PS than before PS (Bonferroni-corrected *p* = 0.032) at SF 6. This indicates that the brain adaptability in AD patients deteriorates with disease progression.

### 3.5. Negative Correlations between MSE Values and EEG Power Ratios in Non-AD Groups

**Figure 6:**
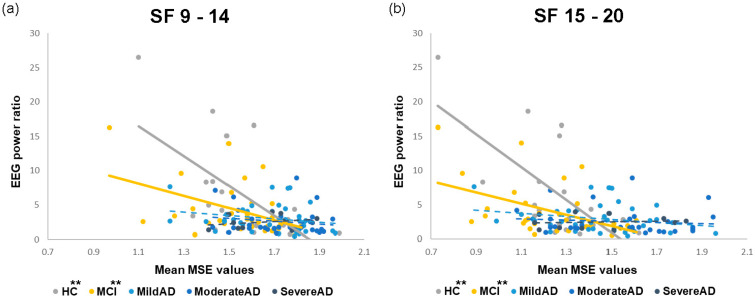
Correlations between mean MSE values at large scale factors and EEG power ratios of 30-Hz harmonic responses to 15-Hz PS at electrode O1: (**a**) SF 9–14 and (**b**) SF 15–20 (** *p* < 0.01).

Only the EEG power ratios and MSE values that presented significant group effects during PS were included in the correlation analysis. This included the EEG power ratios of 30-Hz harmonic responses to 15-Hz PS in all channels of interest (Table 2; Figure 6) as well as the EEG power ratios of 36-Hz harmonic responses to 9-Hz PS in channels O2 and P4 (Table 3). This also included MSE values at large scale factors (one mean MSE value for SF 9–14 and one for SF 15–20). Overall, significant correlations were observed only in the HC and MCI groups, and most of these were in visual areas (electrodes O1 and O2).

In the HC group, mean MSE values over large scale factors were significantly and negatively correlated with the EEG power ratios of 30-Hz harmonic responses in all channels of interest and with the EEG power ratios of 36-Hz harmonic responses at electrodes O2 and P4. In the MCI group, mean MSE values over large scale factors were significantly and negatively correlated with the EEG power ratio of 30-Hz harmonic responses at electrodes O1 and O2.

No significant correlations were observed between mean MSE values and EEG power ratios in any of the AD subgroups, except for a slightly negative correlation between mean MSE values at SF 15–20 and EEG power ratio of 30-Hz harmonic responses at the O2 electrode in the Mild AD group. Note that in the Severe AD group, mean MSE values at SF 9–14 were significantly and positively correlated with the EEG power ratio of 36-Hz harmonic responses at the O2 electrode (Table 3). This correlation was opposite to that observed in non-AD groups.

## 4. Discussion

To the best of our knowledge, this is the first study to address perceptual functioning together with brain adaptability before, during, and after intermittent photic stimulation (PS) in patients with AD (mild, moderate, and severe AD), patients with MCI, and healthy elderly controls. This study combined linear spectral analysis (power) and nonlinear dynamical analysis (MSE) to analyze EEG signals obtained during PS. MSE analysis was also used to analyze resting-state EEG signals obtained before and after PS in order to characterize PS-induced changes in brain dynamics. The HC and MCI groups presented similar patterns in neural oscillatory responses and brain dynamics. In contrast, all AD subgroups exhibited diminished neural oscillatory responses and highly irregular brain dynamics during PS. A “transitional change” between MCI and Mild AD group was further revealed by brain dynamics. The AD subgroups did not present the PS-induced changes (Pre-PS vs. During PS vs. Post-PS) in EEG complexity at large time scales observed in the non-AD groups. In the AD subgroups, the associations between EEG complexity and oscillatory responses during PS were also disrupted. Taken together, these findings suggest that brain adaptability, as revealed by PS-induced changes in EEG complexity, is deficient in AD patients, indicating an inability to adapt to repetitive perceptual stimulation and the diminished changes following stimulation.

### 4.1. Diminished EEG Harmonic Responses in AD Patients during Repetitive PS 

Estimating fundamental and harmonic oscillatory responses to repetitive perceptual stimulation is crucial to understanding the perceptual functioning of the brain. We estimated EEG power ratios to various PS frequencies (5-Hz, 9-Hz, and 15-Hz PS) in patients with Mild AD, Moderate AD, Severe AD, and MCI as well as healthy controls. We observed no significant between-group differences in EEG oscillatory responses to any of the fundamental PS frequencies. This suggests that AD patients preserve basic perceptual functioning in their oscillatory responses to repetitive stimulation. Nonetheless, EEG harmonic responses were lower in AD patients than in healthy elderly controls (Figure 2 and Figure 3), consistent with previous findings [49]. The subdued neural oscillatory responses to PS observed in AD patients may stem from a disruption of the thalamo-cortico-thalamic interaction, which has been shown to play a critical role in developing and maintaining neural oscillatory responses to PS [58]. Researchers have previously suggested that the neurotransmitter acetylcholine suppresses the thalamocortical system, resulting in enhanced EEG synchronization [59]. Kikuchi et al. [60] demonstrated that using scopolamine to block the activity of acetylcholine reduced photic-driving neural responses, particularly in occipital regions. We therefore speculate that the diminished harmonic responses to repetitive visual stimuli may be attributed to a disruption of the thalamocortical system that results from a deficiency in acetylcholine in the brains of AD patients [61]. Future studies could further investigate the effects of cholinergic intervention in AD [62,63,64,65] based on treatment-related changes in EEG biomarkers [42,66,67].

### 4.2. Irregular Long-Range Brain Dynamics in AD Patients

Entropy can be used to model the dynamics and adaptability of the brain in response to dynamic environmental stimulation and/or stressors in everyday life [25]. In the current study, nonlinear MSE analysis was used to quantify EEG complexity. We investigated group differences before, during, and after PS separately (Figure 4). Similar group differences were observed before and during PS; group differences were mostly absent after PS.

For resting-state EEG complexity before PS (Figure 4, left columns), at small scale factors, the Severe AD group presented lower resting-state MSE than the non-AD and Mild AD groups. Our results are in line with previous findings that EEG complexity at small time scales was lower in AD and MCI patients than in healthy controls, which may represent regular brain activity and reflect a local loss of complexity in AD patients [14,34]. At large scale factors, in contrast, AD subgroups presented higher resting-state MSE than non-AD groups. In particular, MCI patients could be differentiated from Mild AD patients based on Pre-PS resting-state MSE at large scale factors. After repetitive stimulation (Figure 4, right columns), group differences in resting-state MSE were mainly absent, which might be due to the rebound of brain dynamics in non-AD groups. This will be further discussed together with the within-group PS-induced changes (see Section 4.3).

During PS (Figure 4, middle columns), group differences at large scale factors were similar to those before PS. However, group differences at small scale factors disappeared, which might stem from the increased MSE values to PS (with respect to Pre-PS) in AD subgroups. At large scale factors, the MSE values were significantly higher (i.e., more irregular) in AD subgroups than in non-AD groups. This is an indication that the perceptual system in AD patients is unable to adapt to repetitive stimuli to yield synchronous brain oscillations. This phenomenon has previously been described as frequency-following responses [68], photic-driving responses [69], and steady-state visual evoked potentials (SSVEPs) in response to PS [70]. Cao et al. [71] provided solid evidence that inherent fuzzy entropy values were significantly higher in migraine patients than in healthy controls. Note that migraine sufferers are particularly vulnerable to difficulties in adapting to repetitive visual stimulation. We therefore speculate that in AD patients, a disturbance in neural oscillatory responses to repetitive visual stimuli hinders EEG synchronization to PS, manifesting as brain activity of more significant irregularity during PS.

EEG complexity at different time scales could be a reflection of brain dynamics at different spatial scales. Specifically, brain dynamics at large time scales may reflect brain activities at low frequencies and long-distance functional connectivity [16,17], though counterintuitive representations of frequency correspondence of MSE time scales were recently discussed [72]. Researchers have previously reported a positive correlation between fundamental or harmonic responses (the SSVEP) and global efficiency in healthy individuals, which may be an emergent phenomenon associated with long-range connectivity between the parietal-occipital and frontal regions [46,73]. In the current study, this long-distance connectivity is disrupted in AD patients due perhaps to the accumulation of amyloid plaques and neurofibrillary tangles [18]. From this, it is possible to infer that in AD patients, the high MSE values observed at large scale factors during PS can be attributed to a disruption in long-range brain dynamics.

### 4.3. Diminished PS-Induced Changes in EEG Complexity in AD Patients

PS-induced changes in EEG complexity are crucial to the understanding of brain dynamics in response to repetitive perceptual stimulation and the brain’s ability to revert/rebound to resting-state complexity after a period of stimulation. In this study, PS-induced changes in MSE revealed an interesting pattern that varied as a function of disease severity, as follows: HC > MCI > Mild AD > Moderate AD > Severe AD (Figure 5). This loss of MSE differences among before, during, and after repetitive stimulation in AD subgroups was observed over a wide range of time scales, indicating a breakdown in nonlinear dynamical characteristics of the brain and suggests that the adaptability of the brain in response to perceptual stimulation diminishes with disease progression. 

In the HC and MCI groups, resting-state EEG complexity significantly increased after PS at large time scales, showing strong brain adaptability and an excessive compensatory rebound to resting-state brain dynamics. However, the same effects were absent in the three AD subgroups. The information processing capacity of the brain can be estimated from the complexity of neural signals [25], which is largely due to the transitions between brain states [17,74]. Neuronal degeneration can disrupt structural pathways and long-distance connectivity in the brain (see Section 4.2), thereby reducing the complexity of the neural systems and the ability of the brain to adapt [25,75]. As in previous studies, we observed in AD patients an absence of PS-induced changes in EEG complexity at large time scale factors, due perhaps to a lack of transitioning from the state induced by repetitive visual stimulation to the resting state and a deterioration of long-range connectivity. 

In the AD subgroups, PS-induced changes were observed only at small scale factors. Researchers have previously suggested that EEG complexity at small time scales reflects brain dynamics at high frequencies, stemming from information processing in local brain regions [16]. In all AD subgroups, the MSE values were higher at small scale factors during PS than in the resting states (Pre-PS and/or Post-PS). This may be an indication of a failure to entrain to repetitive visual stimulation or excessive visual information processing locally in the visual cortex. In the Mild and Moderate AD groups, the resting-state MSE values at small scale factors dropped to the lowest level after PS (compared to Pre-PS and During PS), due perhaps to an excessive compensatory reduction in local neural excitability when attempting to recover to resting-state brain complexity from repetitive photic stimulation. In the Severe AD group, PS-induced changes were mostly absent. This observation supports our earlier speculation that the local preservation of brain adaptability in patients with mild and moderate AD is lost in patients with severe AD as the disease progresses. Taken together, our findings support the notion that deficient brain adaptability in AD patients is an indication of the brain becoming “stuck in a rut”.

### 4.4. Disrupted Association between EEG Complexity and Neural Oscillatory Responses during PS in AD Patients

We sought to gain a more comprehensive view of the association between linear brain activities and nonlinear brain dynamics in response to repetitive visual stimulation by evaluating the relationship between MSE values and EEG power ratios during PS in each group (Figure 6; Table 2 and Table 3). We observed significant negative correlations between MSE values and EEG power ratios in the HC and MCI groups but not in the AD subgroups. Moreover, the negative correlation coefficients in the HC group were higher than those in the MCI group. These findings again corroborate the idea that when an intermittent, rhythmic perceptual stimulation is presented at a constant frequency (such as PS at a specific frequency), a healthy and robust brain system has the ability to adapt to or to show habituation to that repeated stimulation [76]. Gradual synchronization and regularization of brain activity increase photic-driving oscillatory responses to that frequency and decrease dynamic complexity. Researchers have previously demonstrated that SSVEP responses were positively correlated to long-range cortical connections [46]. Thus, it is possible that in the current study, the diminished correlation between brain complexity and neural oscillatory responses during PS in AD patients can be attributed to a disruption of long-range cortical connectivity.

### 4.5. Similar Neural Oscillatory Responses and Brain Complexity in HC and MCI Groups

MCI patients are at an elevated risk of progressing to AD. One interesting finding in the current study was that neural oscillatory responses and brain complexity in the MCI group were indistinguishable from those in the HC group but clearly discernible from those in the AD subgroups (Figure 2, Figure 3 and Figure 4). Likewise, researchers have previously reported similar patterns in HC and MCI individuals [37,77,78]. One FDG-PET study reported that in AD-like MCI patients, metabolism in the left PCC and precuneus was lower than in non-AD-like MCI patients [78]. One longitudinal EEG study reported that alpha power was lower in MCI patients who converted to AD than in stable-MCI patients [77]. 

Interestingly, the MCI group, on the one hand, presented increased complexity after PS at large scale factors that were similar to the HC group; on the other hand, the MCI group presented decreased complexity after PS at small scale factors similar to the AD subgroups (Figure 5). This shows that PS-induced brain dynamics could reveal the “transitional change” between MCI and mild AD patients. Future research should focus on brain dynamics and adaptability in AD-like vs. non-AD-like MCI patients. Our findings revealed no evidence of deterioration in the basic perceptual functions in MCI patients; however, further research will be required to ascertain whether this extends to higher cognitive functions as well.

### 4.6. Limitations

This study had a number of limitations, which should be considered in the interpretation of the results. First, this study was based on routine clinical EEG examinations, in which participants were recruited from outpatient settings in the hospital. As stated in Section 2.1, subjects underwent clinical diagnosis by neurologists and were screened according to rigorous inclusion/exclusion criteria. Subjects who had significant neurological diseases other than AD or had a history of significant head trauma followed by permanent neurological deficits or known structural brain abnormalities were excluded. Due to potential ethical and medical issues, participants were not asked to stop their regular medications or routine treatments before the EEG examination. Therefore, we cannot rule out the possibility that AD and MCI patients were suffering from other comorbidities or were under the effects of medication used, such as acetylcholinesterase inhibitors [79,80]. Future studies could estimate cholinergic treatment-related changes in EEG biomarkers in AD as reported in previous studies [42,66,67].

Second, all patients were clinically diagnosed by neurologists and underwent various neuropsychological examinations and assessments, and our results revealed statistically significant differences between the Severe AD group and the other groups. Nonetheless, the small number of Severe AD patients (*N* = 11) means that caution must be exercised in generalizing the results pertaining to this group. 

Third, age was considered an important covariate in studying the aging population. Furthermore, AD severity is a continuum rather than a discrete, independent, categorical factor. To avoid exploratory and complex analysis, this study conducted planned comparisons using one-way ANCOVAs with age as a covariate (to test between-group MSE differences at each condition) and one-way repeated measures ANOVAs (to test PS-induced changes in each group) with Bonferroni comparisons. For future brain network analysis, two-way mixed ANCOVAs for each time scale factor, with group as the between-subject factor, PS condition as the within-subject factor, and age as the covariate, would be a comprehensive analysis. 

Fourth, this study investigated brain activity in response to photic stimulation in a clinical setting with a particular focus on EEG electrodes related to the visual cortex, which precluded comprehensive source-level analysis. In the future, it may be possible to use high-density EEG to gain a more comprehensive understanding of the neural sources and underlying neural mechanisms. 

Fifth, this study used sample entropy [12] as the entropic metric of multiscale entropy analysis [10,34] to quantify EEG complexity in AD, MCI patients, and elderly controls, as widely adopted by clinical studies investigating MSE-based nonlinear analysis of EEG signals in AD [14,33,34,35,36,37,39,40,41]. This allows straightforward comparisons of the MSE results and for discussing their interpretations related to AD. It should be noted that there are other new entropic metrics and modified variants in the MSE family (see [25,81,82,83,84,85,86] for recent review articles), such as multiscale dispersion entropy [87,88], multiscale fuzzy entropy [89,90], and multiscale fluctuation dispersion entropy [91]. Each has respective methodological assumptions and principles, strengths and advantages, and shortcomings and limitations, as recently well-discussed by Azami et al. in [85]. Nevertheless, the ultimate goal and application scenario of our current photic stimulation‒EEG‒MSE study in AD is to apply a practical and simple tool (that is, photic stimulation) that targets the changes in brain adaptability for rapid assessment of AD and MCI in clinical or outpatient settings. Sample entropy, which is relatively robust to noise [12] and has been one of the most popular entropic measures in quantifying brain signal variability, was therefore adopted in this study. It would be beneficial for future studies to exploratorily quantify the aforementioned entropic metrics in MSE-based nonlinear analysis as complexity measures, for example, using a recently published comprehensive open-access MATLAB toolbox CEPS [86], to thoroughly study and compare different estimations of brain complexity.

## 5. Conclusions

This study investigated neural oscillatory responses and brain dynamics induced by intermittent photic stimulation in patients with MCI, mild AD, moderate AD, and severe AD as well as healthy elderly individuals. EEG power spectral analysis in conjunction with MSE analysis revealed similar neural oscillatory responses and brain dynamics in patients with MCI and healthy elderly controls. AD patients demonstrated diminished neural oscillatory responses and brain dynamics with higher irregularity to repetitive visual stimulation. AD patients also demonstrated a notable absence of changes in brain dynamics after repetitive stimulation. Our findings suggest that in addition to memory and cognitive dysfunction, AD may also be correlated with the deterioration in the ability of the brain to respond to perceptual stimulation and revert to the resting state. Interestingly, a “transitional change” between MCI and mild AD patients could be revealed by PS-induced brain dynamics. Early detection of the conversion from MCI to AD may be helpful in providing appropriate treatments for MCI patients and preventing the development of severe AD. In the future, brain oscillatory responses and brain dynamics induced by PS could potentially be used as a tool for the rapid screening of AD.

## Figures and Tables

**Table 1:** Demographic data and MMSE results in the five study groups.

	HC (*N* = 20)	MCI (*N* = 34)	Mild AD (*N* = 38)	Moderate AD (*N* = 31)	Severe AD (*N* = 11)	*p* Values	Post-Hoc Comparisons * (with Bonferroni Correction)
Age (y)	71.00 ± 5.63	68.38 ± 6.49	75.13 ± 8.81	77.87 ± 8.50	72.64 ± 11.54	<0.001 *	Moderate AD > HC Moderate AD > MCI Mild AD > MCI
Gender (Male%)	50%	44%	66%	45%	36%	0.254	-
Education (y)	11.90 ± 4.38	11.89 ± 3.77	11.09 ± 4.42	8.65 ± 3.80	8.73 ± 4.92	0.007 *	MCI > Moderate AD
MMSE	28.15 ± 1.57	26.82 ± 1.91	21.47 ± 1.27	17.29 ± 1.53	10.18 ± 3.74	<0.001 *	HC ≈ MCI > Mild AD > Moderate AD > Severe AD

Data are presented as mean ± SD (* *p* < 0.05). AD, Alzheimer’s disease; HC, healthy elderly control group; MCI, mild cognitive impairment group; MMSE, mini-mental state examination; SD, standard deviation.

**Table 2:** Spearman’s correlation coefficients between mean MSE values at large scale factors and EEG power ratios of 30-Hz harmonic responses to the 15-Hz PS.

SF (Electrode)	HC	MCI	Mild AD	Moderate AD	Severe AD
SF 9–14 (O1)					
*rho*	−0.621	−0.441	−0.240	−0.229	0.182
*p*	0.003 **	0.009 **	0.146	0.215	0.593
SF 15–20 (O1)					
*rho*	−0.644	−0.516	−0.216	−0.195	0.082
*p*	0.002 **	0.002 **	0.193	0.293	0.811
SF 9–14 (O2)					
*rho*	−0.621	−0.437	−0.289	0.033	0.396
*p*	0.003 **	0.010 **	0.079	0.859	0.228
SF 15–20 (O2)					
*rho*	−0.520	−0.487	−0.324	−0.096	−0.050
*p*	0.019 *	0.003 **	0.047*	0.606	0.884
SF 9–14 (P3)					
*rho*	−0.568	−0.165	−0.173	−0.083	0.136
*p*	0.009 **	0.35	0.298	0.656	0.689
SF 15–20 (P3)					
*rho*	−0.442	−0.125	−0.038	−0.203	0.136
*p*	0.051	0.48	0.821	0.273	0.689
SF 9–14 (P4)					
*rho*	−0.496	−0.291	−0.175	0.059	0.236
*p*	0.026 *	0.094	0.294	0.751	0.484
SF 15–20 (P4)					
*rho*	−0.262	−0.213	−0.161	−0.060	−0.018
*p*	0.265	0.226	0.336	0.747	0.958

Electrodes are listed in brackets after scale factors (* *p* < 0.05, ** *p* < 0.01). AD, Alzheimer’s disease; HC, healthy elderly control; MCI, mild cognitive impairment; MSE, multiscale sample entropy; PS, photic stimulation; SF, scale factor.

**Table 3:** Spearman’s correlation coefficients between mean MSE values at large scale factors and EEG power ratios of 36-Hz harmonic responses to 9-Hz PS.

SF (Electrode)	HC	MCI	Mild AD	Moderate AD	Severe AD
SF 9–14 (O2)					
*rho*	−0.534	−0.224	−0.147	−0.125	0.627
*p*	0.015 *	0.203	0.380	0.501	0.039 *
SF 15–20 (O2)					
*rho*	−0.498	−0.178	−0.187	−0.244	0.336
*p*	0.026 *	0.313	0.261	0.186	0.312
SF 9–14 (P4)					
*rho*	−0.600	−0.251	−0.005	0.026	0.182
*p*	0.005 **	0.152	0.975	0.891	0.592
SF 15–20 (P4)					
*rho*	−0.586	−0.388	0.037	−0.096	−0.073
*p*	0.007 **	0.023 *	0.825	0.608	0.831

Electrodes are listed in brackets after scale factors (* *p* < 0.05, ** *p* < 0.01). AD, Alzheimer’s disease; HC, healthy elderly control; MCI, mild cognitive impairment; MSE, multiscale sample entropy; PS, photic stimulation; SF, scale factor.

## Data Availability

The data presented in this study are available on request from the corresponding author. The data are not publicly available due to ethics and privacy.

## Supplementary Materials

The following are available online at https://www.mdpi.com/article/10.3390/e23040427/s1. Figure S1: MSE profiles and between-group differences in MSE values during 5-Hz PS. Figure S2: MSE profiles and between-group differences in MSE values during 9-Hz PS.
