# Supplementary figures and images for: Brain Dynamics Altered by Photic Stimulation in Patients with Alzheimer’s Disease and Mild Cognitive Impairment
Source: Entropy (Basel). 2021 Apr 4;23(4):427. doi: 10.3390/e23040427 (PMC8066899; doi:10.3390/e23040427)

# 5-Hz PS

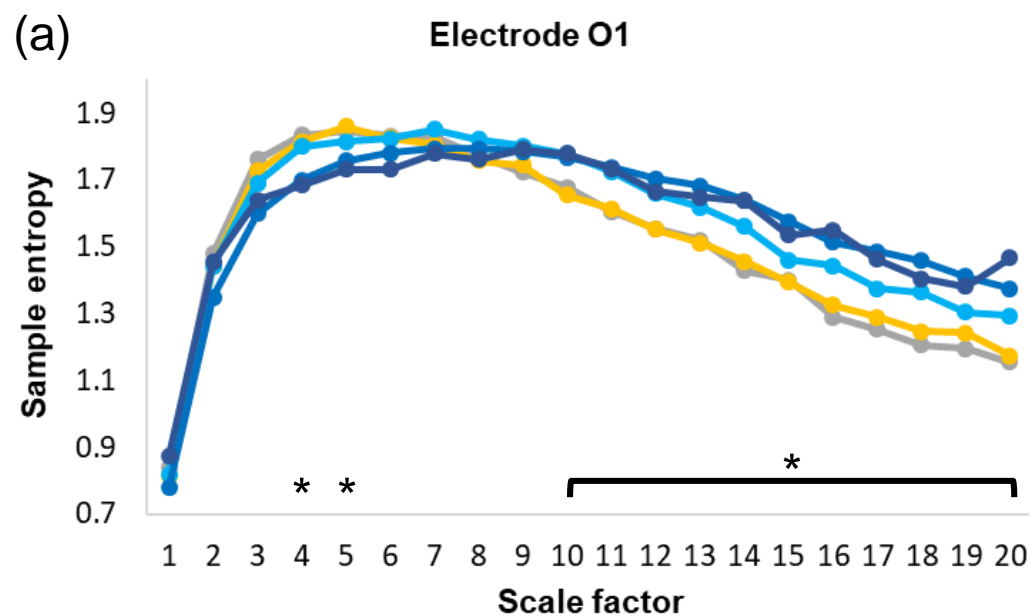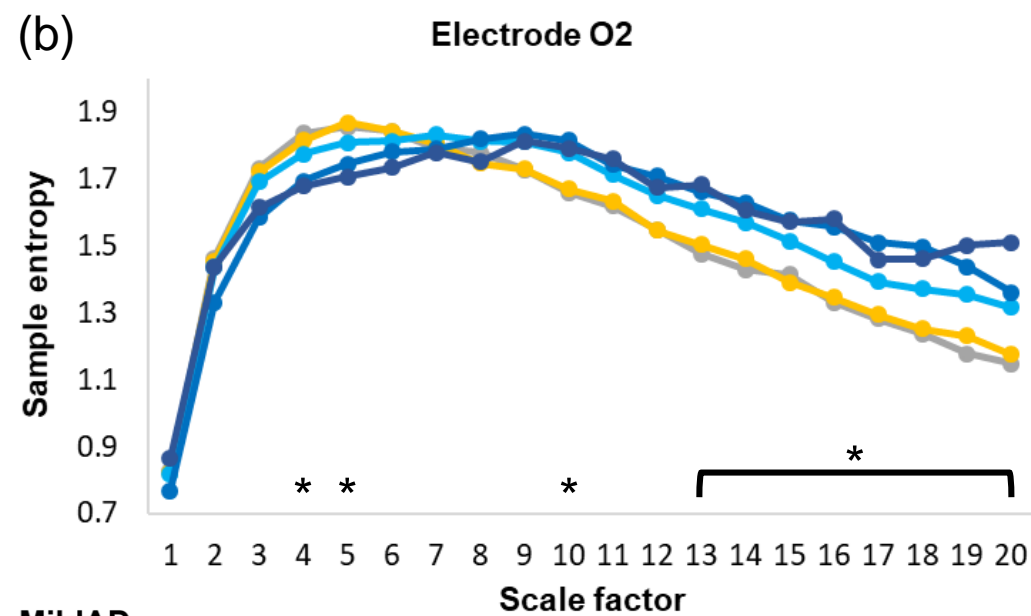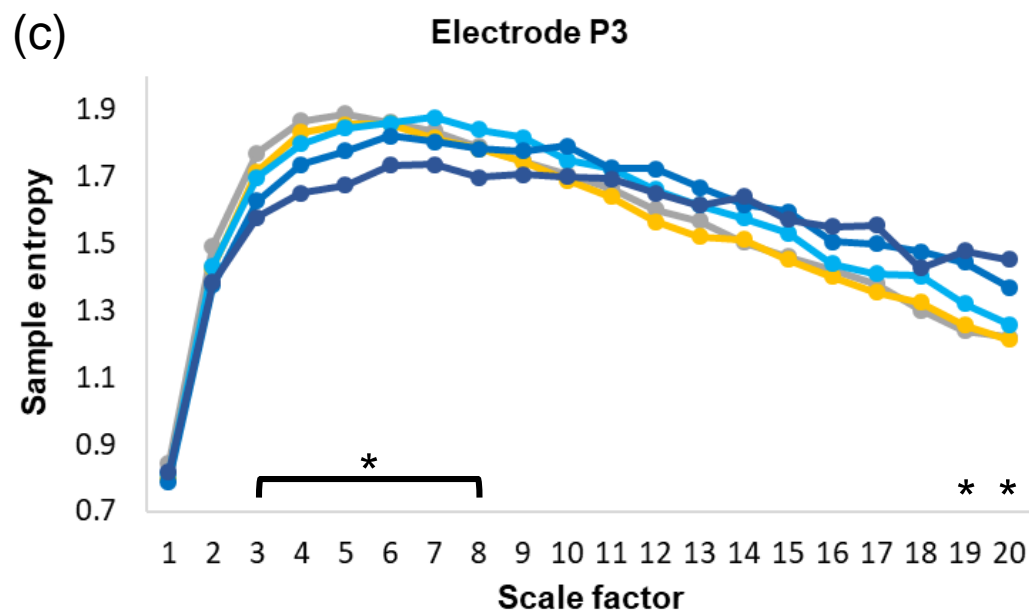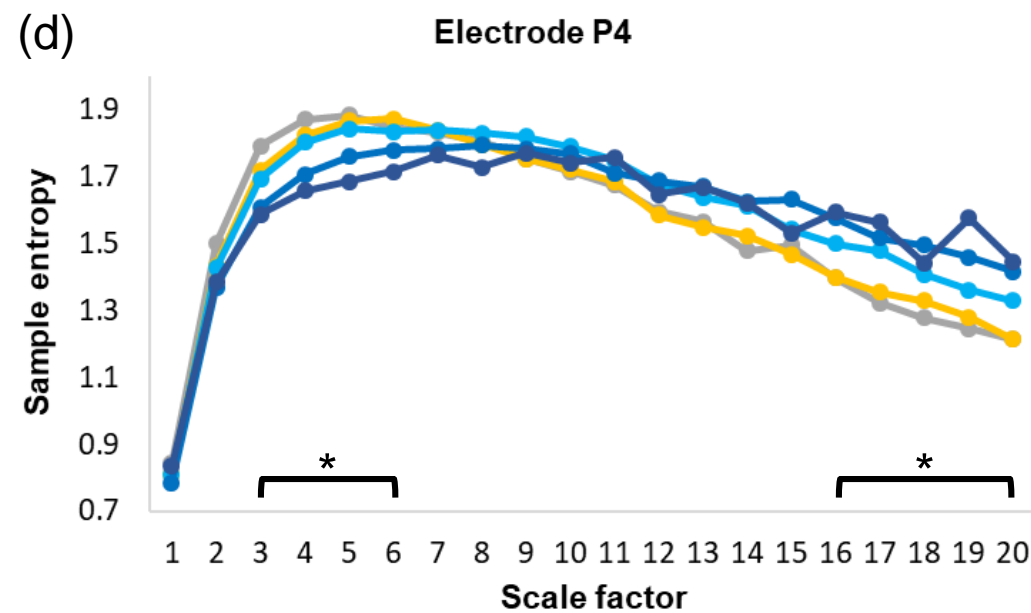

Supplement: Supplementary file 1 [file entropy-23-00427-s001.zip › Supplementary_Figures_Submitted/Figure_S1_Between-group differences in MSE during 5-Hz PS.pdf]

# 9-Hz PS

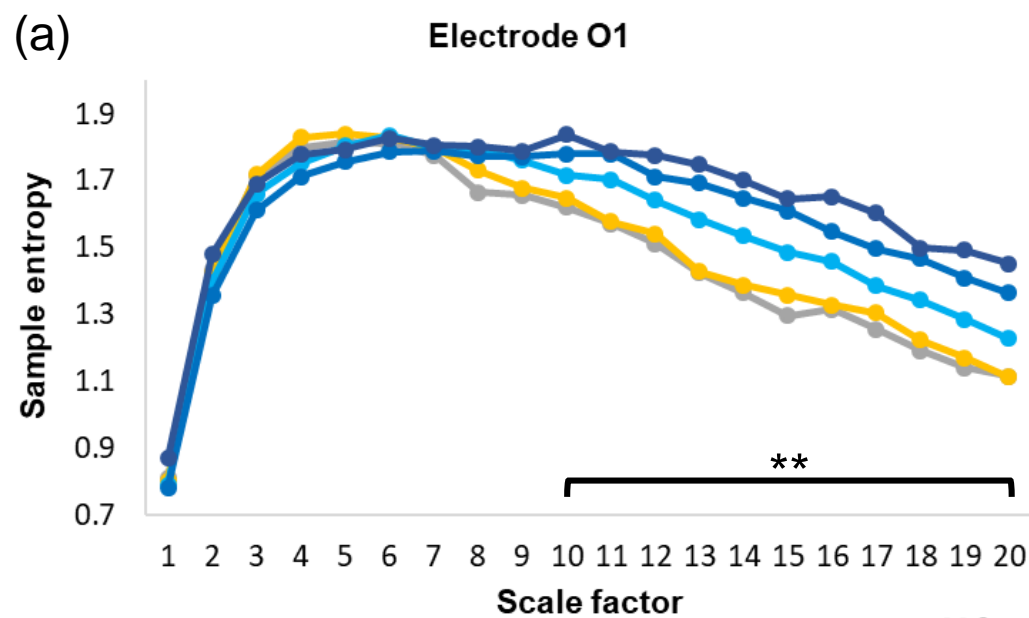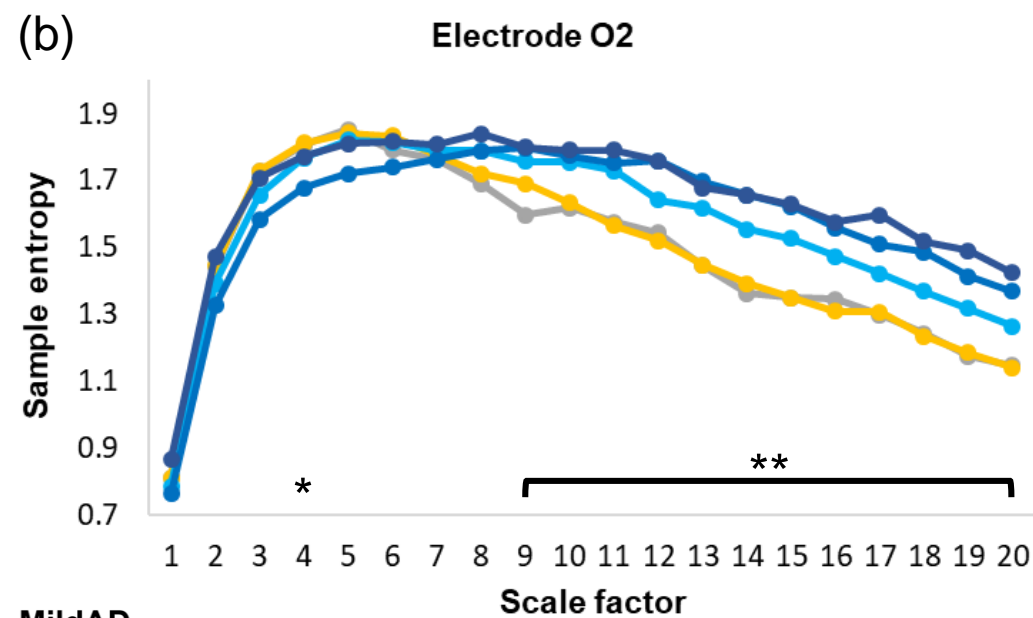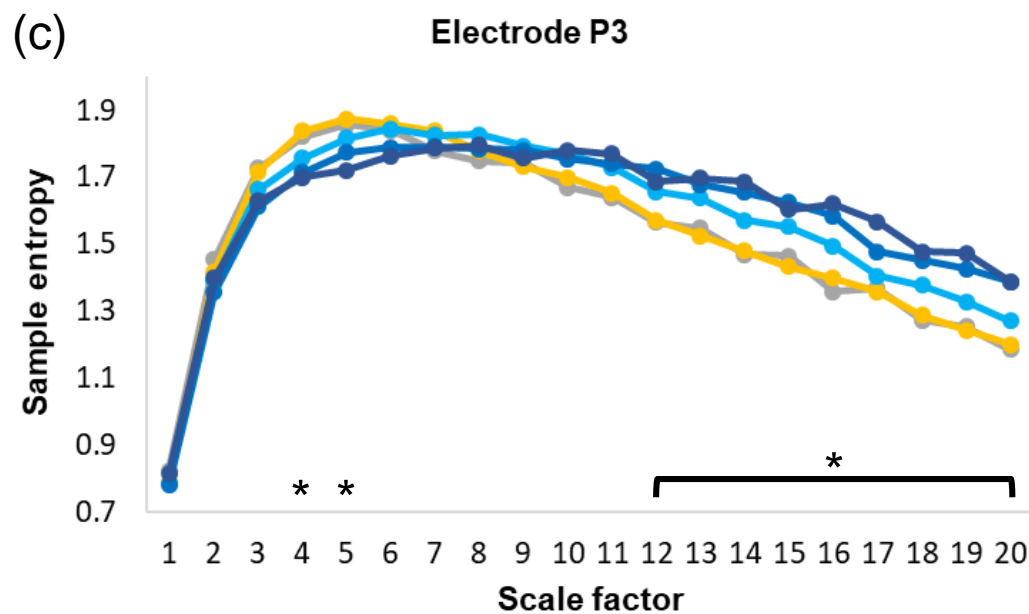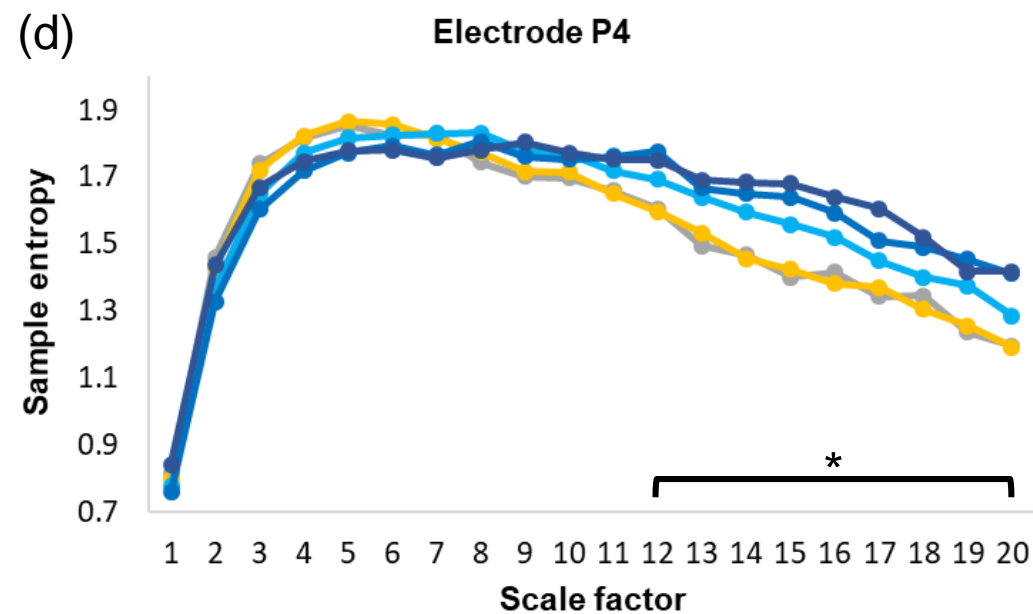

Supplement: Supplementary file 1 [file entropy-23-00427-s001.zip › Supplementary_Figures_Submitted/Figure_S2_Between-group differences in MSE during 9-Hz PS.pdf]
